# Comparison of somatic mutation calling methods in amplicon and whole exome sequence data

**DOI:** 10.1186/1471-2164-15-244

**Published:** 2014-03-28

**Authors:** Huilei Xu, John DiCarlo, Ravi Vijaya Satya, Quan Peng, Yexun Wang

**Affiliations:** 1 Research and Foundation Department, QIAGEN Sciences, Inc., Frederick, MD, USA

## Abstract

**Background:**

High-throughput sequencing is rapidly becoming common practice in clinical diagnosis and cancer research. Many algorithms have been developed for somatic single nucleotide variant (SNV) detection in matched tumor-normal DNA sequencing. Although numerous studies have compared the performance of various algorithms on exome data, there has not yet been a systematic evaluation using PCR-enriched amplicon data with a range of variant allele fractions. The recently developed gold standard variant set for the reference individual NA12878 by the NIST-led “Genome in a Bottle” Consortium (NIST-GIAB) provides a good resource to evaluate admixtures with various SNV fractions.

**Results:**

Using the NIST-GIAB gold standard, we compared the performance of five popular somatic SNV calling algorithms (GATK UnifiedGenotyper followed by simple subtraction, MuTect, Strelka, SomaticSniper and VarScan2) for matched tumor-normal amplicon and exome sequencing data.

**Conclusions:**

We demonstrated that the five commonly used somatic SNV calling methods are applicable to both targeted amplicon and exome sequencing data. However, the sensitivities of these methods vary based on the allelic fraction of the mutation in the tumor sample. Our analysis can assist researchers in choosing a somatic SNV calling method suitable for their specific needs.

## Background

Somatic point mutation calling from matched tumor-normal patient samples is a critical step for cancer genome characterization and clinical genotyping [1,2]. Over the last few years, next-generation sequencing (NGS) has become a popular strategy for genotyping, enabling more precise mutation detection compared to traditional methods due to its high resolution and high throughput [3]. Nevertheless, detecting somatic mutations is still challenging, especially for low-allelic-fraction variants caused by tumor heterogeneity, copy number alteration, and sample degradation [4-6]. Errors in base calling and read alignment present additional challenges for achieving sensitive and specific somatic variant calls. In recent years, several methods have been developed to enhance somatic mutation calling accuracy [7-11]. In general, these methods belong to two families: (1) independent analysis for tumor and normal datasets from an individual followed by SNV type classification using a statistical significance test or simple subtraction (e.g. [12]); (2) simultaneous analysis for matched tumor and normal datasets using joint probability-based statistical approaches, e.g. SomaticSniper [9] and Strelka [11]. In general, agreement among different algorithms is relatively low [13,14], making selection of candidate SNVs for further validation difficult. This disagreement is likely partially due to different error models and prior assumptions underlying each algorithm.

In light of these challenges, there is a need to systematically evaluate the performance of different SNV calling algorithms to help guide best practices in the research community. Recently, several independent studies compared somatic SNV calling tools [15-17]. For example, Roberts et al. [15] compared the performance of VarScan, SomaticSniper, JointSNVMix2, and Strelka in detecting SNVs from matched cancer-normal Illumina exome sequencing of a chronic myeloid leukemia patient. They reported a large difference in called variant sets among algorithms. In a separate study, Wang et al. [17] evaluated 6 tools (EBCall, JointSNVMix, MuTect, SomaticSniper, Strelka, and VarScan2) on tumor-normal pairs using both whole-genome sequencing and exome sequencing data. According to their evaluation, MuTect achieved the highest sensitivity for calling low-allelic-fraction somatic variants. Most of these studies utilized publically available exome data from hybridization capture enrichment, and some used additional validation data from alternative technologies. Complimentarily, Spencer et al. [16] assessed SAMtools, Genome Analysis Toolkit (GATK), VarScan2, and SPLINTER in detecting low-allelic-fraction variants from synthetic mixtures of targeted hybridization capture sequencing reads. They reported that VarScan2 achieved the highest sensitivity but with a high false positive rate, whereas SPLINTER yielded both high sensitivity and specificity.

Exome capture sequencing is typically limited to ~60x read depth (due to cost), and therefore using exome reads limits the potential to detect low-allelic-fraction SNVs in the sample, especially those at less than 5%. Detecting low-allelic-fraction SNVs in the sample is often important for early diagnosis, prevention of drug resistance, and detection of residual tumors. Targeted panel sequencing has been widely used to increase SNV detection sensitivity by achieving a much higher median read depth (>500x), even on a bench top sequencer [18,19]. Among the different enrichment methods available for targeted sequencing, PCR amplicon-based enrichment has been widely popular for small- to medium-sized gene panels due to its low input requirement, simple protocol, fast turnaround time, and good performance on FFPE samples. While there are differences between read properties from PCR amplicon sequencing and exome sequencing, many of which could affect SNV calling, there has not yet been a detailed comparison of SNV calling algorithms using reads from PCR amplicon sequencing. In addition to higher read depth, reads from PCR amplicons have more uniformly defined ends, more PCR duplication, and may contain more amplification bias. Because many variant calling algorithms were developed using whole genome or exome data, the question remains of whether performance differs when these algorithms are applied to amplicon sequencing data.

Another challenge in comparing variant calling methods is to identify a set of true positive and true negative variants in the sample, ideally at various allele fractions. This task is not straightforward, due to the lack of sufficient available validation data. One standard assessment method is to check the overlap between variant sets as a way to assess the concordance and discrepancies among different calling methods. Alternatively, some researchers have randomly chosen a few candidates for Sanger sequencing or used SNP arrays for validation. However, Sanger sequencing involves high cost and labor, making the generation of large-scale validation data difficult. In addition, some of the low-allelic-fraction SNVs are very difficult to validate by Sanger sequencing or SNP array due to the detection sensitivity of these technologies (typically insufficient for variants below 20% fraction). To overcome these limitations, the NIST-led “Genome in a Bottle” Consortium (NIST-GIAB) [20] developed a community resource of high-confidence variants for the reference individual NA12878. From the July 17, 2013 data release, the NIST-GIAB standard is an integration of 12 datasets generated using 5 different sequencing platforms, with the reads aligned using 7 different aligners, and variants called using 3 different variant callers. The availability of the NA12878 reference sample (from Coriell Institute) along with a high-confidence variant set facilitates empirical evaluation of all steps in NGS analysis: enrichment, library preparation, sequencing, alignment, and variant calling. The NIST-GIAB NA12878 variant standard is the basis of emerging tools such as GCAT (http://www.bioplanet.com/gcat), which provides a community platform for comparing variant calling approaches.

In order to evaluate the performance of popular SNV callers on targeted amplicon sequencing reads, especially for low-allelic-fraction SNVs, we mixed reference DNA from NA12878 with DNA from another 1,000 Genomes Project sample (NA19129) at various ratios, thus creating virtual tumor-normal samples with NA12878 “tumor” variant frequencies ranging from 4% to 100%. We then subjected the dilution series samples to multiplex PCR-based enrichment and Illumina MiSeq sequencing. Here we present a comparison of 5 somatic SNV callers using targeted amplicon sequencing from these virtual tumor-normal samples against the NIST-GIAB gold standard variant set. To explore possible performance differences of the SNV callers on amplicon versus exome sequencing data, we also did a similar *in silico* dilution series analysis using publicly available exome data from NA12878. Our results demonstrate the strengths and weaknesses of each method in calling somatic SNVs at diverse levels of allelic fraction, and also the value of the newly established NIST-GIAB gold standard in facilitating such comparisons.

## Results

### Comparison of somatic point mutation calling methods in the benchmark amplicon sequencing data

**Figure 1:**
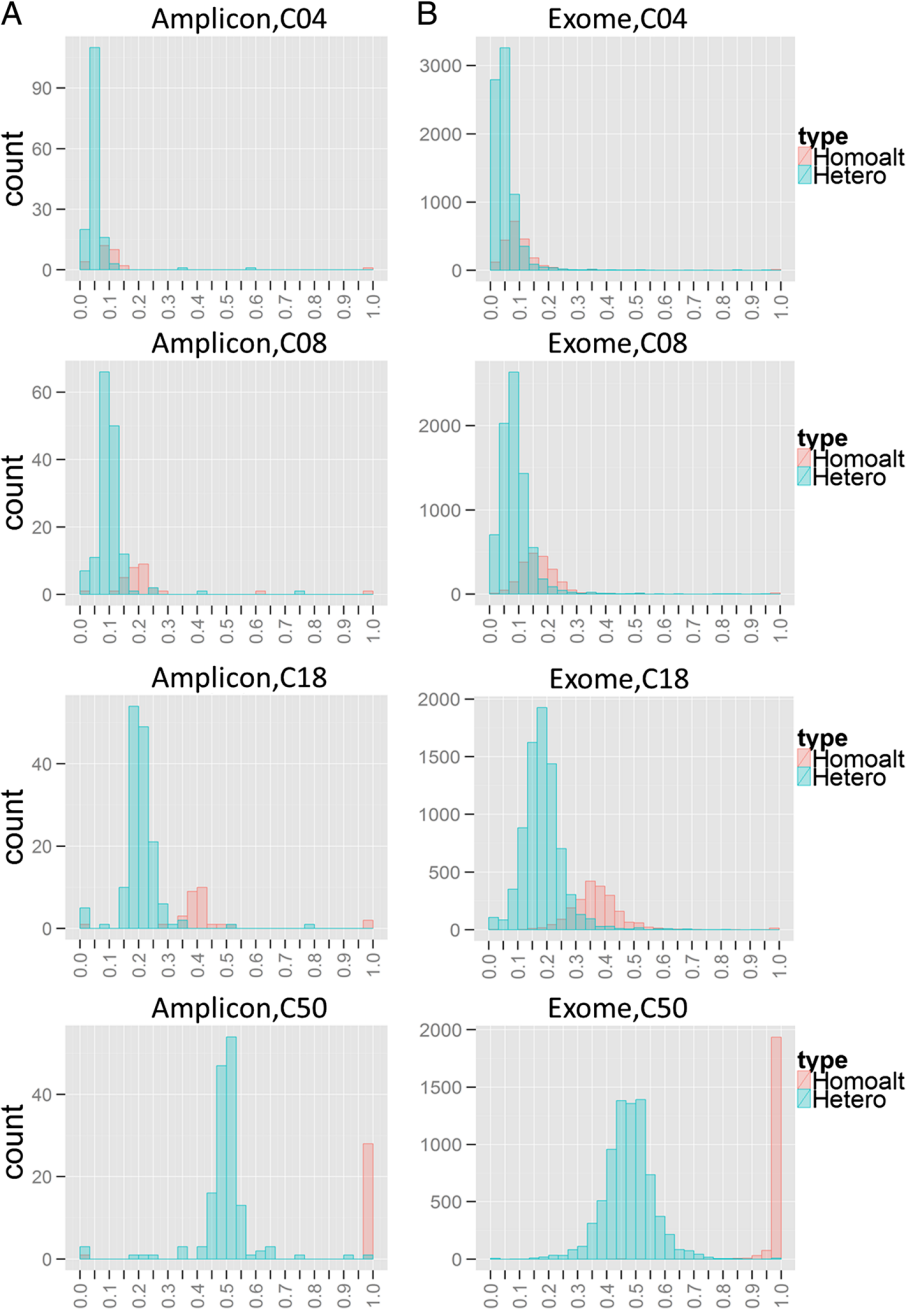
**Distribution of variant allele fraction of NA12878 SNV sites.** Distribution of NA12878 unique SNV sites allele fraction of **(A)** amplicon sequencing experimental dilution series of one replicate over the CCP region of interest and **(B)** exome sequencing *in silico* dilution series over the exome region of interest. The x-axis represents the variant allele fraction and the y-axis represents the number of sites. Homozygous alternate alleles in NA12878 are shown in red, and heterozygous alternate alleles are shown in blue.

Our goal was to evaluate the performance of somatic SNV detection methods from matched tumor-normal samples using amplicon sequencing. For this purpose, we generated a dilution series of samples with 0%, 8%, 16%, 36%, and 100% NA12878 mixed in the control NA19129, to mimic production of artificial tumor sample paired with the 100% NA19129 background as the artificial normal sample. Because the majority of variants in the gold standard are heterozygous (the ratio of heterozygous to homozygous alternative variants is 5 to 1), the main allele fractions we evaluated are 4%, 8%, 18%, and 50%. Therefore, we labelled the samples as C04 (4% as the expected heterozygous variant allele), C08 (8%), C18 (18%), and C50 (50%) in the following descriptions and results. We used QIAGEN’s GeneRead DNAseq Comprehensive Cancer Gene Panel (CCP, Version 1) for enrichment and library construction in triplicate [21]. Then we sequenced the samples for the 124 CCP genes using an Illumina MiSeq sequencer. The median coverage for each triplicate was between 500x and 700x. First, we examined the observed minor allele fraction of NA12878 “somatic” variants to verify the mixture concentration (Figure 1A). Generally, both heterozygous and homozygous alternate allele frequencies fell within the expected range. To systematically evaluate variant calling methods, each read set was subjected to the same alignment and preprocessing steps before variant calling (see Methods). The resulting BAM files were then used as input to five somatic mutation calling methods, including (1) MuTect, (2) GATK UnifiedGenotyper [22] followed by simple subtraction (or simply referred as “NaiveSubtract” in this article), (3) SomaticSniper, (4) Strelka, and (5) VarScan2 (Methods and Table 1). All methods are open-source algorithms except for the “NaiveSubtract” method, which calls SNVs by identifying the variants present in the “tumor” sample VCF file but not in the “normal” sample VCF file. The variants set for “tumor” and “normal” samples were called independently using GATK UnifiedGenotyper.

**Table 1:** List of somatic SNV calling methods and versions

**Methods**	**Version**	**Reference**
GATK UnifiedGenotyper in NaiveSubtract	v2.3.9	[22]
MuTect	v1.1.4	[7]
SomaticSniper	v1.0.2	[9]
Strelka	v1.0.7	[11]
VarScan2	v2.3.6	[8]

**Figure 2:**
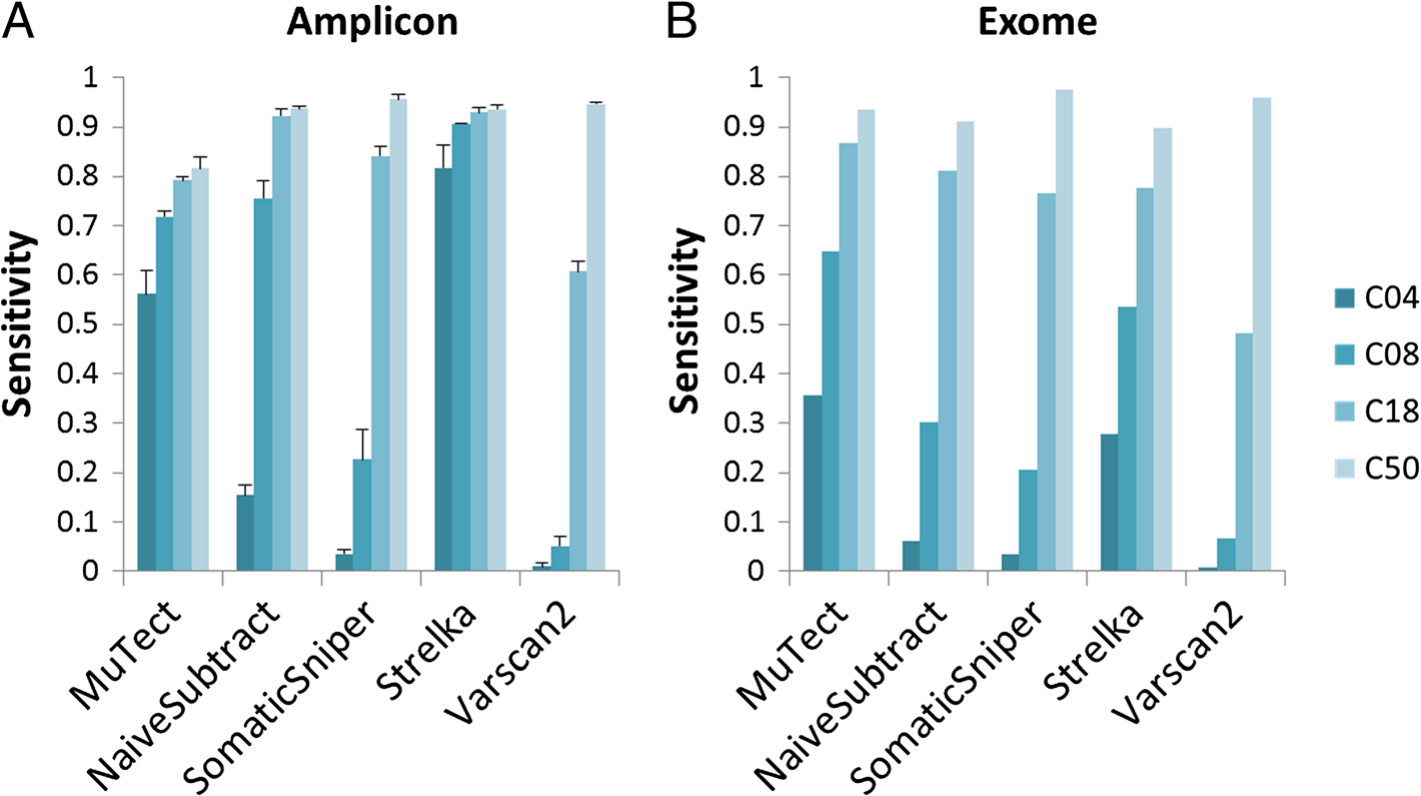
**Sensitivity of somatic SNV calling methods.** The x-axis represents the methods and the y-axis represents sensitivity for **(A)** amplicon sequencing data and **(B)** exome sequencing data. C04, C08, C18, and C50 represent mixing concentrations of 8%, 16%, 36%, and 100% samples in amplicon sequencing data, and median allele fractions of 4%, 8%, 18%, and 50% for NA12878 unique heterozygous SNVs in *in silico* mixture exome sequencing data over the region of interest. For the amplicon sequencing data, columns represent the mean and error bars represent the standard deviation of the triplicate.

**Figure 3:**
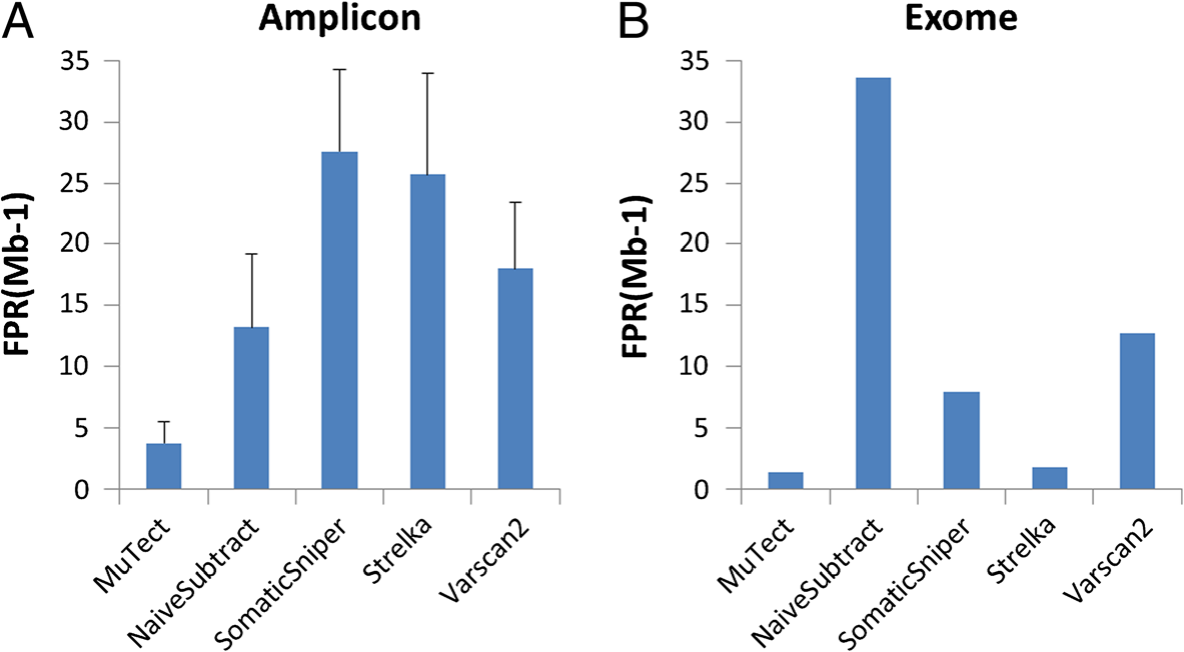
**Specificity of somatic SNV calling methods.** The x-axis represents the methods and the y-axis represents false positives per Mb over the region of interest, for **(A)** the amplicon sequencing dilution series triplicate (mean?±?standard deviation); **(B)** exome sequencing 100% NA12878 sample.

We assessed the sensitivity and specificity of each variant calling algorithm on each virtual tumor sample using the NIST-GIAB gold standard variant set (Version 2.15). Our analysis region excludes sites where NA19129 is not a homozygous reference (for region of interest (ROI), see Additional file 1: Table S1). We compared somatic SNV calling performance at various “tumor purity” levels (Figure 2A). At the lowest purity level, the highest sensitivity was achieved by Strelka (0.851?±?0.05) under the settings recommended by its authors. MuTect, with its default setting under HC mode (high-confidence mode), achieved lower sensitivity than Strelka (about 0.60), although this might in part be due to MuTect being tuned for higher specificity. In terms of specificity, because we expect that the number of false positives detected does not depend on the fraction of NA12878 in the “tumor” sample, we calculated the false positive rate by treating each single virtual tumor sample as a replicate (12 total replicates). MuTect produced the lowest false positive rate, whereas the other four methods provided similar levels of specificity (Figure 3A). Overall, Strelka and MuTect are capable of detecting candidate somatic SNVs at sites of low allelic fraction. This property is of key importance for studying cancer subclones and highly heterogeneous tumor samples. The sensitivity of all methods increased in samples with a larger fraction of NA12878. As shown in Figure 2A, all of the methods perform fairly well for calling sites in the 100% NA12878 sample. The impact of algorithm selection became weaker for high-allelic-fraction somatic SNV calling. VarScan2, among other traditional approaches, calls variants from each sample independently to estimate the difference between control and disease samples. VarScan2 did not perform well for calling low-allelic-fraction variants. Though VarScan2 can achieve a higher sensitivity of 0.5 if its minimum allele fraction threshold is lowered to 0.05, it then generates a much higher false positive rate (300 false positives per Mb). For SomaticSniper, examination of the distribution of somatic scores (data not shown) indicates that a cut-off of 20 can reduce false positives without compromising sensitivity. Therefore, we added an ad hoc post-calling filtering step with a somatic score cut-off of 20. Nevertheless, SomaticSniper gave low sensitivity in low-allelic-fraction variant calling, even though it achieves the highest sensitivity for the 100% “pure tumor” sample. As for NaiveSubtract, our results indicate that GATK UnifiedGenotyper can detect very few variants at <8% allelic fraction. Therefore, when used with PCR amplicon sequencing data with a median read depth above 500x, Strelka and MuTect can call variants at all allelic fraction levels, whereas NaiveSubtract, VarScan2, and SomaticSniper provide less sensitivity for calling somatic SNVs of low allelic fraction, at least when using their default settings. Consistent with [15], the statistical model-based methods such as MuTect and Strelka are more efficient, because they consider the correlations between tumor-normal pairs and calculate the joint probabilities of the genotype pairs.

### Trade-offs between sensitivity and specificity for MuTect and Strelka

**Figure 4:**
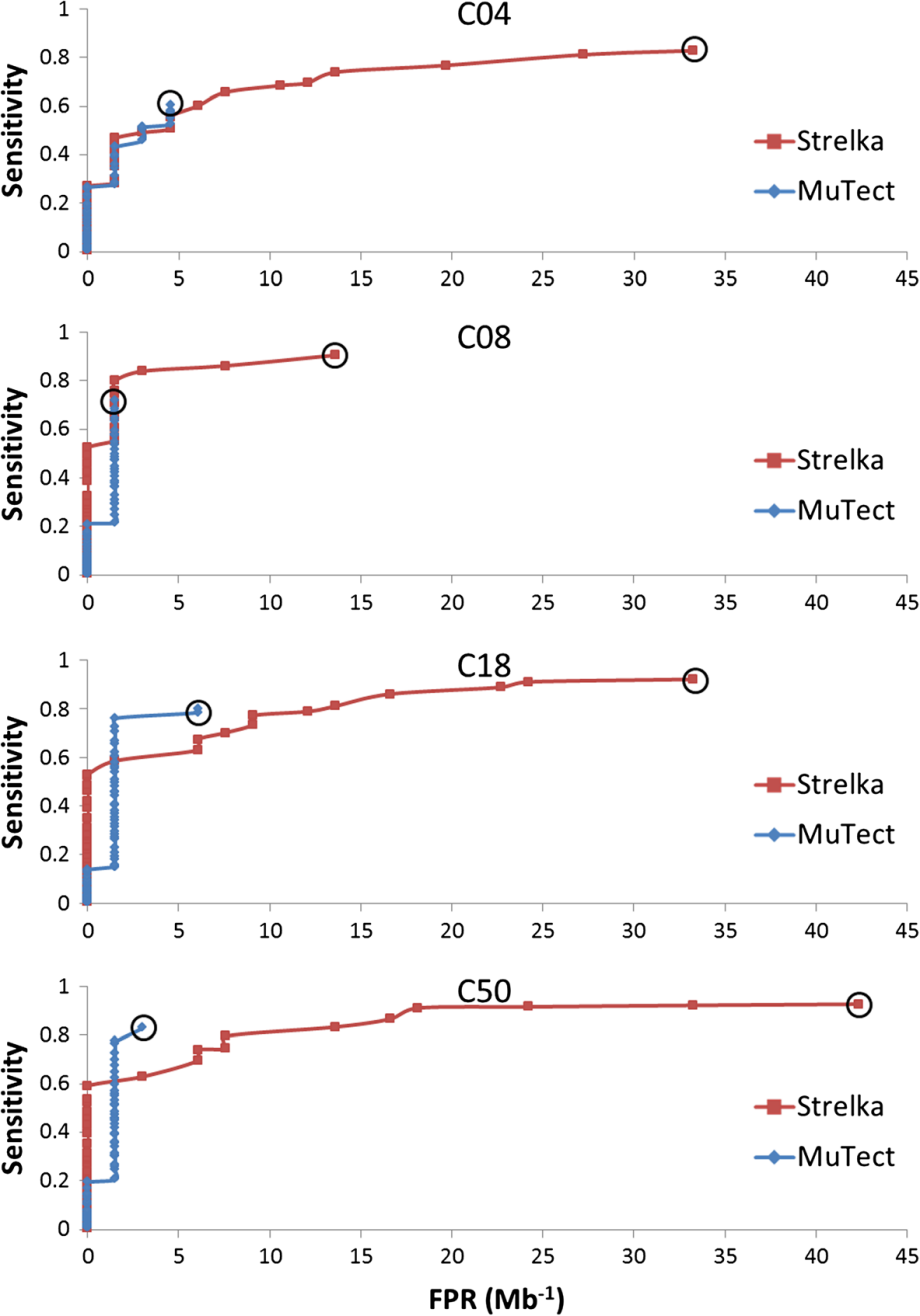
**ROC-like curves summarizing sensitivity and specificity of MuTect and Strelka.** Sensitivity and FPR (per Mb) plots using various values of MuTect LOD threshold and Strelka QSS_NT threshold generated from one dilution series replicate of amplicon sequencing data. Original thresholds in each final model are marked with black circles (corresponding to outputs in Figure 2).

Next we further examined the difference between MuTect and Strelka. We chose to focus on these two methods out of the five because they achieved significantly higher sensitivity at the lowest SNV fraction with similar or lower false positive rates than the other methods. Previous studies have compared the performance of MuTect and Strelka in exome sequencing [7]. As shown in Figures 2A and 3A for our amplicon sequencing, Strelka achieved significantly better sensitivity than MuTect under the recommended settings, but at the expense of a much higher false positive rate. The question remains whether this difference would disappear by further tuning the algorithms or it represents a true difference in performance. We examined the performance trade-offs by varying cut-offs for the MuTect somatic variant log-likelihood score and the Strelka quality score, which reflect the joint probability of a somatic variant and a normal genotype (Figure 4). While differences exist between the two methods when using default settings, we observed that they can achieve very similar sensitivity/specificity trade-offs for lower SNV fractions (C04 and C08) by tuning the core statistics parameter and/or post-calling filters. However, the disparity between the methods remains at higher SNV fractions (C18 and C50), where MuTect can achieve higher sensitivity with a lower false positive rate. One possible explanation for this continuing difference is the sampling limitation for C04 and C08. Low-allelic-fraction SNVs may not be sampled sufficiently during enrichment and sequenced with adequate read depth, potentially limiting our ability to assess performance differences in C04 and C08. Alternatively, more sequencing errors and dimers may be called as variants at greater abundance levels. Overall, our analysis demonstrated that the algorithms differ in sensitivity and specificity, and users should therefore select their algorithm depending on which performance characteristic is the highest priority.

### Comparison of somatic point mutation calling methods in exome sequencing data

Because most matched tumor-normal variant callers were developed using exome sequencing data, we next assessed whether their respective performance differs between high-read-depth PCR-based amplicon sequencing and low-read-depth hybridization capture-based exome sequencing. The two approaches have different read depths and amplification bias, which could affect variant calling accuracy. Therefore, we compared the abilities of the aforementioned five methods to accurately call somatic SNVs in exome sequencing data. For exome reads, we chose a different Yoruba individual (NA18489) than we used for amplicon sequencing (NA19129), because exome sequencing reads for NA18489 were generated using the same capture procedure used for NA12878. The exome sequencing data of NA12878 and NA18489 are publicly available, as part of the 1,000 Genomes Project (Additional file 1: Table S2). We downsampled the preprocessed BAM files and, *in silico*, mixed the resulting subsets of reads to desired “tumor” sample purities. The resulting *in silico* mixture series is diluted in the same way as the amplicon sequencing experimental dilution series: at NA12878 unique heterozygous (homozygous alternative) variant positions over the region of interest, the median variant allele fraction is 4% (8%), 8% (16%), 18% (36%) and 50% (100%), respectively (Figure 1B). Then we applied the five variant calling methods on the *in silico* matched “tumor-normal” exome sequencing pairs over the region of interest (Additional file 1: Table S1). Additionally, we checked performance results from the CCP region of the exome sequencing data and verified that there are no special advantages/disadvantages to the CCP region relative to the exome capture region (data not shown).

Overall, the sensitivity ranking of each method is comparable with what was achieved in targeted amplicon sequencing data (Figure 2). For the exome data, MuTect and Strelka detected more somatic mutations than the other methods. MuTect gave the best sensitivity among lower concentrations and Strelka gave the second-best sensitivity. Results from the MuTect publication [7] show that for variants at 5% abundance, MuTect’s sensitivity was between 0.16 and 0.53 for sequencing depth between 30x and 60x, whereas Strelka, used with standard settings, detected between 0.16 and 0.40 in the same sequencing depth range. Therefore, our present observations are consistent with this previous study. SomaticSniper, VarScan2, and NaiveSubtract still yielded low sensitivity in detecting lower-allelic-fraction somatic SNVs. In addition, consistent with what was observed in the targeted sequencing data, all methods achieved high sensitivity in the high-allelic-fraction tumor samples.

We next assessed specificity using only the C50 sample in order not to introduce potential artifacts arising from the *in silico* mixing. MuTect and Strelka achieved the highest specificity (Figure 3). All methods except NaiveSubtract showed a lower false positive rate compared to amplicon sequencing. This is could be partially due to the fact that all the methods were primarily designed on genome-wide sequencing data or exome data and therefore better control false positives in that data type. Furthermore, since false positive rate is positively correlated with sequencing read depth [7], the greater number of reads generated by amplicon sequencing technology may yield more false positives.

## Discussion

As the first step in analyzing cancer sequencing data, detecting variants with high sensitivity and specificity is of great importance. Our results highlight the differences in performance between five algorithms when used with targeted amplicon versus exome sequencing data. When analyzing different types of data, use of different algorithms may be appropriate. For example, methods using a hypothesis of Poisson/binomial distribution of alternate allele read depth would deviate from actual exome sequencing data, which display a notable overdispersion in the distribution of alternate allele read depth [23]. In terms of sensitivity, we observed that the five algorithms perform better in calling low-allelic-fraction variants from amplicon sequencing data than from exome data, probably due to the higher sequencing depth in the former. In fact, when the amplicon sequencing data were *in silico* downsampled to the same sequencing depth as the exome data, the sensitivity from all algorithms dropped and declined more significantly in C04 and C08, while the relative performance ranking remained the same (data not shown). On the other hand, since sequencing read depth is among the factors affecting false positive rate, most algorithms generate more false positives in amplicon sequencing data. However, we also demonstrate through Strelka and MuTect that further tuning the “joint probability” score away from the default setting can reduce the false positives in amplicon sequencing to levels similar to that for exome sequencing, without substantially compromising sensitivity. In summary, the relative performance ranking among five variant callers examined in hybridization capture-based exome sequencing largely holds true for PCR-based amplicon sequencing. Amplicon sequencing can achieve much higher variant detection sensitivity for low-allelic-fraction SNVs. At the same time, default variant calling settings should be further tuned to dampen false positive rates when analyzing amplicon sequencing data.

Regarding individual algorithms, MuTect and Strelka show similarly high sensitivity in detecting low-allelic-fraction SNVs in amplicon sequencing reads, with good specificity. For high-allelic-fraction somatic SNVs, VarScan2 and SomaticSniper perform slightly better than MuTect and Strelka in terms of sensitivity in both amplicon sequencing and exome sequencing data.

NaiveSubtract is very straightforward in concept. Using NaiveSubtract, variants detected in the tumor but not the normal sample would be considered putative somatic mutations. Early studies applied such a subtraction method in detecting somatic mutations and demonstrated success to some extent [12]. One drawback of NaiveSubtract is that it does not take into account the complexity of germline false positives due to PCR-based enrichment artifacts or sequencing errors, or false negatives due to under-sampling during enrichment. Also, NaiveSubtract is not tailored to handle detection of allele sites with low fraction. In other words, the sensitivity is limited by the actual variant caller used, which, in this case, is GATK UnifiedGenotyper.

The performance of VarScan2 is closely related to the minimum allele fraction threshold, because VarScan2 will suppress any mutations below this threshold. We could improve VarScan2 sensitivity after tuning the minimum allele fraction cut-off from the default 0.20 to a lower value, but at the cost of significantly compromised specificity (data not shown). In one recent study [16], VarScan2 achieved a sensitivity of 0.97 for detecting variants of 5% fraction in targeted sequencing with average coverage of >1000×. However, the false positive rate in this study was 59 per Mb after the poor quality, low coverage, and germline sites were masked, which is about 3-fold higher than our results. Furthermore, prior knowledge about tumor purity is unavailable in real-world applications, so users often use default settings to run VarScan2. Using the default VarScan2 settings, we were unable to call somatic SNVs of low allelic fraction.

Although we set the parameters to maximize sensitivity, Somatic Sniper’s performance still falls behind other algorithms in calling somatic SNVs of low allelic fraction. However, SomaticSniper did achieve the highest sensitivity in the 100% ‘pure tumor’-normal match. The low sensitivity of VarScan2 and SomaticSniper in calling low-allelic-fraction somatic SNVs is consistent with the results in [17], which showed sensitivity less than 0.10 for somatic SNVs of fraction lower than 0.20. In contrast, MuTect and Strelka produce high-confidence somatic SNV candidates for any level of allele fraction: MuTect and Strelka can robustly detect somatic SNVs at low allelic fraction, with an average sensitivity of 0.89 and 0.86 for sites above 0.05 allelic fraction, respectively.

The merits of using artificial matched tumor-normal sample mixture series data sets created for calling NA12878 “somatic” SNVs are at least two-fold: (1) the NIST-GIAB gold-standard is a well-defined, high-confidence, large set of SNVs for benchmark evaluation, and (2) this setup allowed us to assess variant calling for somatic SNVs of a wide range of allele fractions from real data. The advantages of the NIST-GIAB gold standard lie in the fact that more sites could be assessed as compared to SNP arrays, which are normally limited to common variants. However, one limitation of our analysis is that a high-confidence variant list for the background germline variants is not available. Therefore, using NA12878 for an artificial “somatic” sample is likely more accurate in evaluating sensitivity than in evaluating specificity. Another limitation is that the NA12878 DNA purchased from Coriell is not exactly the same batch used for the NIST-GIAB variant calls. In addition, one constraint of our artificial matched normal samples is the unmatched error distribution between the “normal” and “tumor” samples. So the virtual tumor-normal matches would potentially reduce the performance of methods relative to performance on real tumor samples that have error distributions more similar to the matched normal sample.

In principle, characterizing how performance of different methods depends on factors such as allele fraction is necessary for designing experiments. The complexity and systematic noise from sequencing from tumor-normal matched samples affect each algorithm, but does so in different ways. For systematic comparison, here we used the same aligned read file for all the methods, whereas impact of aligners on the methods would further distinguish their performance. Deeper understanding of the sample properties and the ultimate goals of a cancer research project will make a difference in the choice of algorithms. This work can be extended to address questions regarding detecting variants from small amount of target genome mixed in the control samples. Our work provides insights for the community to assist in choosing sensitive and specific methods for somatic SNV calling, given a specific set of data type and experimental conditions.

## Conclusions

In this work, we demonstrated that the five somatic SNV calling methods are applicable to both matched tumor-normal targeted amplicon and exome sequencing data. Sensitivities vary with allelic fraction of the mutation in the tumor sample. Our results show MuTect and Strelka achieved the highest sensitivities. Our analysis can assist researchers in choosing a somatic SNV calling method for their specific needs.

## Methods

### Virtual tumor-normal targeted amplicon sequencing data

DNA samples of NA12878 and NA19129 were purchased from Coriell Institute. Sample mixtures were created based on the actual amplifiable DNA in each sample, resulting in 0%, 8%, 16%, 36%, and 100% of NA12878 sample mixed in the NA19129 sample, respectively. We treated the mixed samples at 8%, 16%, 36%, and 100% as the virtual tumor samples and the 0% as the virtual normal sample. Therefore, we produced artificially matched tumor-normal samples to call somatic SNVs. QIAGEN’s GeneRead DNAseq Comprehensive Cancer Gene Panel (Version 1) was used to amplify the target region of interest (124 genes, 800 Kb). Targeted enrichment and library construction were done following manufacturer’s user manual (QIAGEN). Briefly, 160 ng of each virtual tumor sample or virtual normal sample were PCR amplified and purified. For each sample, one quarter of the PCR product was used for constructing a barcoded Illumina DNA library. Libraries were quantified using QIAGEN’s GeneRead DNAseq Library Quant System, and were mixed in equal amount. Illumina MiSeq sequencing was performed following manufacturer’s user manual (Illumina) to generate FASTQ files, resulting in median coverage depth of 500–700x for each sample. Experiments were performed in triplicate. The sequencing reads were aligned to the reference genome using BWA [24], then preprocessed according to Broad best practice guidance (indel realignment, base quality score recalibration, and base alignment quality scoring). Finally, the primer sequences were trimmed away from the pre-processed reads before variant calling.

### Virtual tumor-normal whole exome sequencing data

Whole exome sequencing data of NA12878 and NA18489 were obtained from the 1,000 Genomes Project [25]. Raw reads were mapped to the hg19 human reference genome sequence (GRCh37) by the 1,000 Genomes Project. The publicly available read alignment files in BAM format were generated with BWA mapping, GATK local realignment around known indels, GATK base quality score recalibration, and Picard MarkDuplicates. Each BAM file was randomly downsampled using Picard command-line tools with various percentages to achieve the following mixture concentrations: 8%, 16%, 36%, and 50%. One NA12878 downsampled result and one NA18489 downsampled result were then merged using SAMtools [26]. This step was repeated for different mixture percentages to create an *in silico* dilution series. Consequently, the primary NA18489 sequencing results represent the virtual normal sample and the NA12878 dilution series were treated as the matched virtual tumor samples.

### Region of interest

For targeted amplicon sequencing data, the region of interest (ROI) includes 661,684 bases, resulting from the intersection between QIAGEN CCP panel regions and NIST-GIAB high-confidence callable regions (Version 2.15, excluding genomic sites with conflicting genotype evidence, repetitive regions, decoy sequences, or reported structural variants, as well as regions/variant locations with evidence of bias (systematic sequencing errors, local alignment bias, etc.)), plus masking 213 bp of NA19129 variant sites annotated by 1,000 Genomes Project (ftp://ftp-trace.ncbi.nih.gov/1000genomes/ftp/release/20110521/). For exome sequencing data, the region of interest include 33,886,321 bases, resulting from the intersection between the exome capture regions with at least one read coverage in both samples and the NIST-GIAB high-confidence sites, plus masking 12,974 bp of NA18489 variant sites annotated by 1,000 Genomes Project (ftp://ftp-trace.ncbi.nih.gov/1000genomes/ftp/release/20110521/). For these regions of interest, the NIST-GIAB gold standard contains a 181 and 9,868 ‘somatic’ SNVs for our targeted-sequencing and exome-sequencing evaluation, respectively (for details see Additional file 1: Table S1).

### Variant calling algorithms

Altogether, we included the following methods for comparison (for corresponding command lines for calling the five algorithms, see Additional file 1):

1. NaiveSubtract — SNVs were called separately from virtual tumor and normal samples using GATK UnifiedGenotyper [22]. For exome sequencing data, reads were already mapped, locally realigned and recalibrated by the 1,000 Genomes Project. So SNVs were directly called on the BAM files using GATK Unified Genotyper. Then, SNVs detected in the virtual normal sample were removed from the list of SNVs detected in the virtual tumor sample, leaving the “somatic” SNVs.

2. MuTect — MuTect is a method developed for detecting the most likely somatic point mutations in NGS data using a Bayesian classifier approach. The method includes pre-processing aligned reads separately in tumor and normal samples and post-processing resulting variants by applying an additional set of filters. We ran MuTect under the High-Confidence mode with its default parameter settings. We disabled the “Clustered position” filter and the “dbSNP filter” for the amplicon sequencing reads, and we disabled the “dbSNP filter” for the exome sequencing.

3. SomaticSniper — SomaticSniper calculates the Bayesian posterior probability of each possible joint genotype across the normal and cancer samples. We tuned the software’s parameters to increase sensitivity and then filtered raw results using a Somatic Score cut-off of 20 to improve specificity.

4. Strelka — Strelka reports the most likely genotype for tumor and normal samples based on a Bayesian probability model. Post-calling filters built into the software are based on factors such as read depth, mismatches, and overlap with indels. We skipped depth filtration for exome and amplicon sequencing data as recommended by the Strelka authors. For the amplicon sequencing reads, we set the minimum MAPQ score at 17 for consistency with the defaults in GATK UnifiedGenotyper. We used variants passing Strelka post-calling filters for analysis.

5. VarScan2 — VarScan2 performs analyses independently on pileup files from the tumor and normal samples to heuristically call a genotype at positions achieving certain thresholds of coverage and quality. Then, sites of the genotypes not matched in tumor and normal samples are classified into somatic, germline, or ambiguous groups using Fisher’s exact test. We generated the pileup files using SAMtools mpileup command.

The compatibility of the output VCF files between different methods as well as the NIST-GIAB gold standard was examined using bcbio.variation tools and manual inspection. The reported SNP call representations between files are comparable to each other.

### Performance metrics

We used sensitivity and false positive rate as the performance metrics:

$$\mathrm{Sensitivity}\;\left(\mathrm{aka}.\mathrm{Recall}\right)=\frac{\mathrm{TP}}{\mathrm{TP}+\mathrm{FN}}$$

$$\mathrm{FPR}\;\left(Mb^{-1}\right)\left(\mathrm{False}\;\mathrm{Postive}\;\mathrm{Rate}\;\mathrm{per}\;\mathrm{Mb}\right)=\frac{\mathrm{FP}}{\mathrm{FP}+\mathrm{TN}}\times 10^{6}$$

### Availability of supporting data

The command line scripts of variant calling algorithms we used in the paper are included in the additional files.

## Competing interests

All authors are employees of QIAGEN Sciences. We declare that our employment with QIAGEN did not influence our interpretation of data or presentation of information.

## Authors’ contributions

YW designed the study, managed the project, wrote the paper, and was responsible for the final submission and revisions of the manuscript. HX designed the study, performed various analyses, generated figures and wrote the paper. JD designed the study, managed the project and wrote the paper. RVS performed various analyses. QP performed the experiments and wrote the paper. All authors read and approved the final manuscript.

## Supplementary Material

Additional file 1 Supplementary Tables and Supplementary Methods. Click here for file
