# Supplementary material for: Comparison of somatic mutation calling methods in amplicon and whole exome sequence data
Source: BMC Genomics. 2014 Mar 28;15:244. doi: 10.1186/1471-2164-15-244 (PMC3986649; doi:10.1186/1471-2164-15-244)
Supplement: Additional file 1 — Supplementary Tables and Supplementary Methods. [file 1471-2164-15-244-S1.docx]

**Supplementary Materials**

**Supplementary Table 1. Summary of regions of interest (ROI) and number of variants in (A) amplicon sequencing and (B) exome sequencing.**

(A)

| Description | Number |
| --- | --- |
| Capture bases | 797,556 |
| Masked bases by NIST/GIB | 135,872 |
| **ROI** | **661,684** |
| NA12878 SNVs within ROI | 394 |
| NA19129 SNVs within ROI | 503 |
| **NA12878 unique SNVs within ROI** | **181** |

(B)

| Description | Number |
| --- | --- |
| Capture bases | 46,205,186 |
| Masked bases by NIST/GIB | 10,118,635 |
| Masked bases of 0 coverage | 2,200,240 |
| **ROI** | **33,886,321** |
| NA12878 SNVs within ROI | 22,842 |
| NA18489 SNVs within ROI | 28,489 |
| **NA12878 unique SNVs within ROI** | **9,868** |

**Supplementary Table 2. Summaries of exome sequencing data from the 1,000 Genomes Project.**

| Property | NA12878 | NA18489 |
| --- | --- | --- |
| ftp | http://ftp.1000genomes.ebi.ac.uk/vol1/  ftp/data/NA12878/exome_alignment/ | http://ftp.1000genomes.ebi.ac.uk/vol1/  ftp/data/NA18489/exome_alignment/ |
| Sequencing Center | Broad Institute | Broad Institute |
| Capture Kit | Agilent SureSelect_All_Exon_V2 | Agilent SureSelect_All_Exon_V2 |
| Covered Bases | 46,205,186 | 46,205,186 |
| Library | Illumina HiSeq 2000 | Illumina HiSeq 2000 |
| Insert Size | 220 | 220 |
| Read Length | 2$\times$76 | 2$\times$76 |
| Aligner | BWA | BWA |
| Total Mapped Bases | 17,031,563,064 | 14,694,320,624 |
| Mean Coverage over capture region | 108 | 93 |
| Median Coverage over capture region | 86 | 74 |

**Supplementary methods**

Command lines for calling SNV softwares

Targeted sequencing

**MuTect:**

java -Xmx2g -jar muTect-1.1.4.jar --analysis_type MuTect --reference_sequence ucsc.hg19.fasta --cosmic CosmicAllMuts_v64_260313_noLimit.sorted_wchr.vcf --dbsnp dbsnp_137.hg19.vcf --intervals CCP.bed --input_file:normal vitural.normal.bam --input_file:tumor virtual.tumor.bam --out out_mutect_extended_stats.out --coverage_file out_mutect_extended.wig.txt --vcf out_mutect_extended.vcf --enable_extended_output --downsample_to_coverage 1000

**NaiveSubtract:**

java -XX:DefaultMaxRAMFraction=1 -XX:+UseParallelGC -jar GenomeAnalysisTKLite.jar -T UnifiedGenotyper -L CCP.bed -dcov 2500 -o normal.vcf -I normal.bam --genotype_likelihoods_model BOTH -minIndelFrac 0.2 --min_base_quality_score 17 --standard_min_confidence_threshold_for_calling 30.0 --standard_min_confidence_threshold_for_emitting 30.0 --baq CALCULATE_AS_NECESSARY --baqGapOpenPenalty 30.0 --defaultBaseQualities -1 --validation_strictness STRICT --interval_merging ALL -R ucsc.hg19.fasta -nt 8 1> VariantCall.log 2> VariantCall.log

java -XX:DefaultMaxRAMFraction=1 -XX:+UseParallelGC -jar GenomeAnalysisTKLite.jar -T UnifiedGenotyper -L CCP.bed -dcov 2500 -o tumor.vcf -I tumor.bam --genotype_likelihoods_model BOTH -minIndelFrac 0.2 --min_base_quality_score 17 --standard_min_confidence_threshold_for_calling 30.0 --standard_min_confidence_threshold_for_emitting 30.0 --baq CALCULATE_AS_NECESSARY --baqGapOpenPenalty 30.0 --defaultBaseQualities -1 --validation_strictness STRICT --interval_merging ALL -R ucsc.hg19.fasta -nt 8 1> VariantCall.log 2> VariantCall.log

* Custom scripts were then used for subtracting the variants in the normal.vcf from the tumor.vcf

**SomaticSniper:**

bam-somaticsniper -q 0 -p -F vcf -f ucsc.hg19.fasta tumor.bam nomal.bam somaticsniper.vcf

**Strelka:**

perl configureStrelkaWorkflow.pl --tumor tumor.bam –normal normal.bam –config strelka_config_ampl_custom.ini --ref ucsc.hg19.fasta

cd strelkaAnalysis

make -j 8

**VarScan2:**

samtools mpileup -q 1 -Q 13 -A -B –l CCP.bed -f ucsc.hg19.fasta tumor.bam>tumor.bam.mpileup

samtools mpileup -q 1 -Q 13 -A -B –l CCP.bed -f ucsc.hg19.fasta normal.bam>normal.bam.mpileup

java -jar VarScan.v2.3.6.jar somatic normal.bam.mpileup tumor.bam.mpileup outputprefix --output-vcf 1

Exome sequencing

**MuTect:**

java -Xmx2g -jar muTect-1.1.4.jar --analysis_type MuTect --reference_sequence hs37d5.fa --intervals exome.bed --input_file:normal normal.bam --input_file:tumor tumor.bam --out out_mutect_extended_stats.out --coverage_file out_mutect_extended.wig.txt --vcf out_mutect_extended.vcf --enable_extended_output --downsample_to_coverage 1000

**NaiveSubtract:**

java -XX:DefaultMaxRAMFraction=1 -XX:+UseParallelGC -jar GenomeAnalysisTKLite.jar -T UnifiedGenotyper -L exome.bed -dcov 2500 -o normal.vcf -I normal.bam --genotype_likelihoods_model BOTH -minIndelFrac 0.2 --min_base_quality_score 17 --standard_min_confidence_threshold_for_calling 30.0 --standard_min_confidence_threshold_for_emitting 30.0 --baq CALCULATE_AS_NECESSARY --baqGapOpenPenalty 30.0 --defaultBaseQualities -1 --validation_strictness STRICT --interval_merging ALL -R hs37d5.fa -nt 8 1> VariantCall.log 2> VariantCall.log

java -XX:DefaultMaxRAMFraction=1 -XX:+UseParallelGC -jar GenomeAnalysisTKLite.jar -T UnifiedGenotyper -L exome.bed -dcov 2500 -o tumor.vcf -I tumor.bam --genotype_likelihoods_model BOTH -minIndelFrac 0.2 --min_base_quality_score 17 --standard_min_confidence_threshold_for_calling 30.0 --standard_min_confidence_threshold_for_emitting 30.0 --baq CALCULATE_AS_NECESSARY --baqGapOpenPenalty 30.0 --defaultBaseQualities -1 --validation_strictness STRICT --interval_merging ALL -R hs37d5.fa -nt 8 1> VariantCall.log 2> VariantCall.log

* Custom scripts were then used for subtracting the variants in the normal.vcf from the tumor.vcf

**SomaticSniper:**

bam-somaticsniper -q 0 -p -F vcf -f hs37d5.fa tumor.bam nomal.bam somaticsniper.vcf

**Strelka:**

perl configureStrelkaWorkflow.pl --tumor tumor.bam –normal normal.bam –config strelka_config_bwa_exome.ini --ref hs37d5.fa

cd strelkaAnalysis

make -j 8

**VarScan2:**

samtools mpileup -q 1 -Q 13 -A -B -l exome.bed -f hs37d5.fa tumor.bam>tumor.bam.mpileup

samtools mpileup -q 1 -Q 13 -A -B -l exome.bed -f hs37d5.fa normal.bam>normal.bam.mpileup

java -jar VarScan.v2.3.6.jar somatic normal.bam.mpileup tumor.bam.mpileup outputprefix --output-vcf 1
